# Host and Microbiome Genome-Wide Association Studies: Current State and Challenges

**DOI:** 10.3389/fgene.2018.00637

**Published:** 2019-01-22

**Authors:** Denis Awany, Imane Allali, Shareefa Dalvie, Sian Hemmings, Kilaza S. Mwaikono, Nicholas E. Thomford, Andres Gomez, Nicola Mulder, Emile R. Chimusa

**Affiliations:** ^1^Division of Human Genetics, Department of Pathology, Institute of Infectious Disease and Molecular Medicine, Faculty of Health Sciences, University of Cape Town, Cape Town, South Africa; ^2^Computational Biology Division, Department of Integrative Biomedical Sciences, Institute of Infectious Disease and Molecular Medicine, Faculty of Health Sciences, University of Cape Town, Cape Town, South Africa; ^3^Department of Psychiatry and Mental Health, University of Cape Town, Cape Town, South Africa; ^4^Department of Psychiatry, Faculty of Medicine and Health Sciences, Stellenbosch University, Cape Town, South Africa; ^5^Department of Animal Science, University of Minnesota-Twin Cities, St. Paul, MN, United States

**Keywords:** genome-wide association study, microbiome, microbiome-GWAS, host-genetic, host–microbiome interaction

## Abstract

The involvement of the microbiome in health and disease is well established. Microbiome genome-wide association studies (mGWAS) are used to elucidate the interaction of host genetic variation with the microbiome. The emergence of this relatively new field has been facilitated by the advent of next generation sequencing technologies that enable the investigation of the complex interaction between host genetics and microbial communities. In this paper, we review recent studies investigating host–microbiome interactions using mGWAS. Additionally, we highlight the marked disparity in the sampling population of mGWAS carried out to date and draw attention to the critical need for inclusion of diverse populations.

## Introduction

**FIGURE 1:**
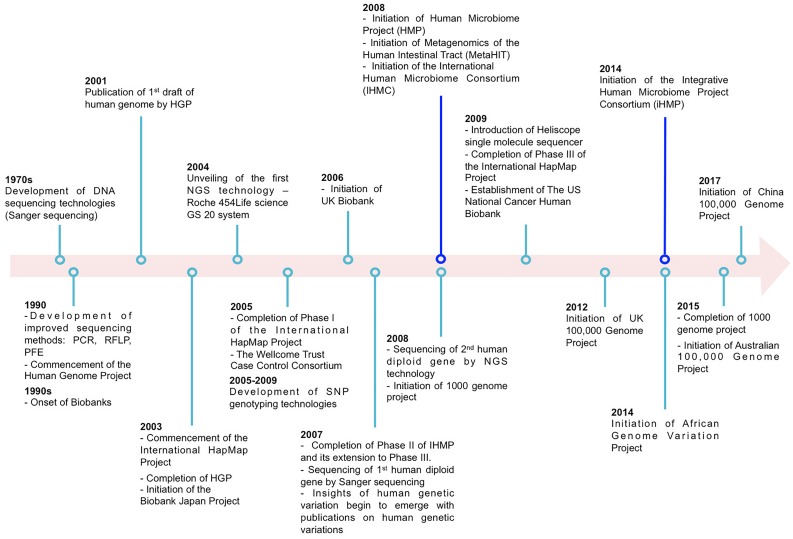
Examples illustrating partial major technological and large-scale collaborative projects (excluding data repositories) on host and microbiome genome-wide association studies (GWAS).

The past two decades have seen tremendous advancement in our understanding of human genetic variation and its implication in health and disease. This has, in part, been facilitated by extensive scientific collaboration and the exponential increase of technical and methodological advancements (Figure 1). Examples of notable large scale scientific collaboration include the Human Genome Project (Sawicki et al., 1993) which published the DNA sequence of the entire human genome; the International Haplotype Map (HapMap) Project (Thorisson et al., 2005) which cataloged the patterns of common polymorphisms (typically minor allele frequency (MAF) larger than 1%) in the human genome and its linkage disequilibrium (LD) structure across multiple ancestral populations. Further, advances in genotyping made feasible, at a relatively low cost, the genotyping of hundreds of thousands (or even millions) of common variants across the human genome. Together these factors catapulted the genome-wide association study (GWAS) in humans (herein referred to as “host”) population.

Genome-wide association study in host populations (hGWAS) has identified hundreds of genetic variants associated with many complex human traits and diseases, novel biological mechanisms and drug targets for infectious and non-infectious diseases (Relling and Evans, 2015). The microbiome, which is the collection of bacteria, archea, fungi, protozoa, and viruses that colonize our body surface and their respective genome (Blum, 2017), has shown to play a major role in human health and disease. The success of hGWAS approach provided an optimistic outlook for and eventual implementation to the microbiome. The microbiome genome-wide association study (mGWAS) aims to identify the host’s genetic polymorphisms that interact with its microbiome. Recently, mGWASs have identified and validated many heritable bacterial taxa, including the *Christensenellaceae* and *Methanogens* families (Goodrich et al., 2017). Moreover, mGWAS has linked host genotypes and identified pathways with inter-individual variability in microbiome composition in states of health and disease (Hall et al., 2017; Imhann et al., 2018). These findings corroborate the common view that the microbiome plays a significant role in a host’s traits, disease susceptibility and resistance, and treatment response.

**FIGURE 2:**
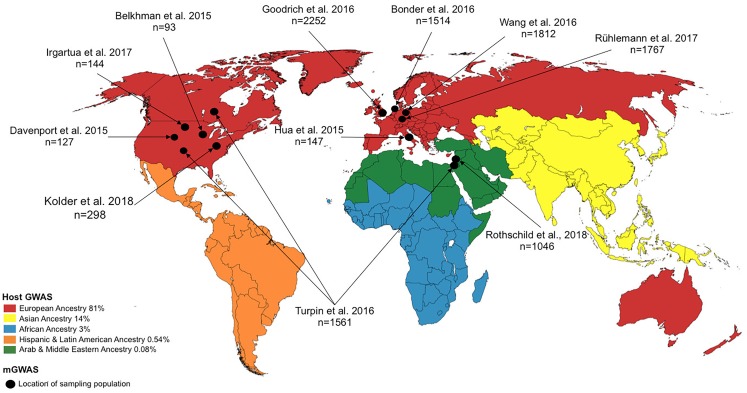
World map showing study location for host GWAS (represented by continent) and microbiome genome-wide association studies (mGWAS) (represented by country/study site). For host GWAS, the data reflects the state of GWAS in 2016 and the locations refers to continental regions and the proportions of host GWAS using samples recruited from those continental regions are as indicated in the legend [data retrieved from Popejoy and Fullerton (2016)]. For mGWAS, the locations refer to the country/study site where the individual for the study were recruited.

Even though multiple lines of evidence have indicated significant host–microbiome interactions (Goodrich et al., 2017; Kurilshikov et al., 2017; Weissbrod et al., 2018), the relative strength of these interactions is unclear, with studies yielding somewhat contrasting results (Rothschild et al., 2018). This is perhaps unsurprising given the plasticity of the microbiome to external factors. In light of this, a key, and yet challenging task, is the establishment of truly causative factors in the observed associations between the environment, host genetics and the microbiome when investigating complex traits and diseases. Including the various microbiome data types, that is, proteomic, metabolomic and transcriptomic, to complement the current mostly used genomic data may help illuminate these interactions. However, combining complex and high dimensional data is not straight forward, introducing yet another challenge. In addition, as in the case of hGWAS (Peprah et al., 2015), the existing disparity in microbiome research, in terms of genomic diversity of the sampling population, further thwarts insights into the complex host–microbiome–environment interaction. As depicted in Figure 2 and elaborated in the section “*disparity in host-microbiome GWAS*,” there is a striking lack of genomic diversity of the study populations in mGWAS published to date.

Here, we review recent studies investigating host–microbiome interactions, through the concept of mGWAS. Then, we highlight the marked disparity in the sampling population of mGWAS carried out to date and draw attention to the critical need for inclusion of non-European populations. Finally, we explore some pertinent challenges in mGWAS.

## Human Variation

In the realm of genetics, human variation, the variation in allele and/or allele frequency, is inherent in all human populations and underlies population differences in many phenotypic expressions, including resistance and susceptibility to diseases. Genomic variation ranges from large microscopic rearrangements such as insertions and deletions, to smaller submicroscopic variations such as single-nucleotide polymorphisms (SNPs) and copy number variation (CNV). Analysis of human genomic variation in the 1000 genomes project reported that a typical genome contains ∼4.1–5.0 million variants, of which >99.9% are SNPs and indels (1000 Genomes Project Consortium et al., 2015). It is important to note, however, that despite being rare (MAF < 0.5%), structural variants affect more bases, have larger effect sizes (Chiang et al., 2017), and are also thought to be potentially involved in disease pathogenesis due to their enrichment for changes that alter protein sequence and function (Casals and Bertranpetit, 2012; Nelson et al., 2012). Thus, in pursuit of unraveling host genetic variants that interact with the microbiome, knowledge of the abundance and distribution of genetic variants along the genome is critical for characterizing the genetic architecture of common as well as rare traits/diseases, and discerning functionally important variations from the myriad of genomic polymorphisms.

With recent technological advances in genotyping arrays and next-generation sequencing (NGS), it is becoming increasingly feasible to conduct large-scale studies, presenting the opportunity for discovery of both rare and common genetic variation. Deep sequencing offers the opportunity to uncover the complete repertoire of these variations (Marth et al., 2011). However, performing deep sequencing on a genome-wide scale is currently limited due to relatively high cost. To this end, whole-exome sequencing and, particularly, genotype arrays have become methods of choice in the geneticist’s arsenal. Intriguingly, these studies, particularly those targeted to protein-coding genes, have revealed the existence of multitude of variants at population and individual level that disrupt protein-coding genes in every human genome (Reijnders et al., 2018), some of which having different phenotypic effects (Blauwendraat et al., 2018; Grarup et al., 2018). These variants generally referred to as loss or gain-of-function variants, occur at low frequency in the genome, and have gene-disrupting ability (MacArthur and Tyler-Smith, 2010); which result in their implications for clinical interpretation of genomic sequences (MacArthur and Tyler-Smith, 2010). It is clear that with NGS technology, many novel genomic variants will be unveiled which, in effect, will facilitate the development of a comprehensive catalog of human genetic diversity.

## Human Microbiome Diversity

The human microbiome exhibits both intra- and inter-individual variability (Lax et al., 2014; Gupta et al., 2017). Studies on twins have shown that the microbiota of identical twins are more similar compared to that of their siblings. Also, siblings have a more similar microbiota than that of unrelated individuals (Goodrich et al., 2014). Similar to genetic variation, the human microbiome plays an important role in health and disease (Blekhman et al., 2015; Hall et al., 2017; Gilbert et al., 2018). The microbiome has been associated with variants in host genes involved in immunity and metabolism (Blekhman et al., 2015). The human immune system has a complex bidirectional relationship with the microbiome. It has been shown that the microbiome is associated with the variability of the immune responses and these responses may also be involved in modifying the microbiome itself (Belkaid and Hand, 2014; Schirmer et al., 2016; Takiishi et al., 2017). Furthermore, human genetic polymorphisms at various loci are hypothesized to interact with each other and with an individual’s microbiome to impact disease (Blekhman et al., 2015; Hall et al., 2017). In particular, mutations in host genes can influence its interaction with the compositional and functional diversity of the microbiome, potentially modulating an individual’s susceptibility to disease (Sandoval-Motta et al., 2017b). In healthy individuals, the microbiome composition is balanced (Rajilic-Stojanovic et al., 2009; Lloyd-Price et al., 2016), and imbalance is now known to be associated with clinical conditions such as diabetes, and inflammatory bowel disease (van Tongeren et al., 2005; Upadhyaya and Banerjee, 2015). Besides host genetics, other factors have been associated with microbial community composition including diet and antibiotic consumption. Many studies have reported that both long- and short-term diet can influence the microbiome composition (Wu et al., 2011; Conlon and Bird, 2014). For example, high-carbohydrate diets have been associated with prevalence of *Prevotella*, while *Bacteroides* are associated with high-fat and high-protein diets (Singh et al., 2017). Additionally, consumption of antibiotics may shift the microbiome composition to a temporally quasi-stable state. This state can be either capable of reverting back to the initial state, or to an alternative irreversible post-antibiotic dysbiosis state (Lozupone et al., 2012). This dysbiosis state is characterized by a loss of taxonomic and functional diversity, which may shift the host’s metabolic capacity and reduce the colonization resistance against invading pathogens (Langdon et al., 2016; Lange et al., 2016).

## Host and Microbiome GWAS

### Host Genome-Wide Association Studies

Genome-wide association study aims to determine the link between genotypic and phenotypic variabilities. This is achieved by obtaining genome-wide genotypic data and phenotypic measurements from a number of subjects, and comparing the frequency of these variants across phenotypic values. GWAS has undoubtedly had successes, identifying thousands of genetic variants associated with hundreds of traits (Visscher et al., 2017), providing valuable insights into the genetic basis of many common traits. Due to LD, any identified associated variant is not necessarily causal as it may simply be “tagging” the causal variant. In addition, most genomic variants are located outside protein-coding regions and are of unknown biological functions (Yang et al., 2017). Consequently, for most traits, little is known about the biological mechanism underlying the associations detected by GWAS.

A critical step toward the elucidation of the underlying biological mechanism is to discern the causal variants. Pinpointing the putative causal variant is, however, challenging for several reasons, including the fact that: (i) most risk regions encompass and implicate multiple variants in the case of complex traits, which without the functional information of the variants, makes it extremely difficult to pinpoint the true causative variant, and (ii) risk variants may reside outside risk regions, and their effects are propagated through regulatory elements (Lin et al., 2016). To this end, several post-GWAS approaches have been introduced (Wang et al., 2010), driven by the need to leverage GWAS summary statistics to account for polygenicity at the SNP, gene or pathway levels to determine the functional role of the identified variants, uncover their biological mode of action and illuminate their regulatory mechanism (Chen et al., 2015; Chimusa et al., 2015).

Also pertinent to host GWAS is the “missing heritability” problem, which describes the observation that the proportion of heritability explained by the GWAS-associated variants is much less than calculated from familial studies. The reasons for this are still unknown and remains controversial (Marian, 2012; Zaitlen and Kraft, 2012; Sandoval-Motta et al., 2017b), with possible reasons being cited to include epistasis, epigenetics, small effect sizes of the variants, poor coverage of genetic variations on genotyping platforms (Hou and Zhao, 2013). Meanwhile, some researchers have attributed this discrepancy to the fact that GWAS only accounts for genetic variation in human cells and does not consider the effects of the microbiome on phenotype (Marian, 2012; Sandoval-Motta et al., 2017a,b). In light of this, incorporating the microbiome into host GWAS has been hypothesized to significantly reduce the missing heritability gap for microbiome-associated traits (Sandoval-Motta et al., 2017b). However, given that it is not yet known why microbiome is more similar in monozygotic than in dizygotic twins, incorporating other potential sources of variability such as diet, behavior – which are usually assumed to be homogenous across the subjects - may help to explain the missing heritability.

### Microbiome Genome-Wide Association Studies: Approaches and Applications

The microbes that inhabit the human body exist in a synergistic relationship with the human host, performing several important roles in metabolism, detoxification, homoeostasis, immunity and epithelial development (Tremaroli and Bäckhed, 2012; Belkaid and Hand, 2014). The human microbiome composition varies widely across different body sites and shows some stability at adulthood for the predominant bacterial communities (Blekhman et al., 2015; Lloyd-Price et al., 2016). Host–microbiome interactions soon establish an equilibrium, which determines the state of health of an individual (Tremaroli and Bäckhed, 2012; Blekhman et al., 2015). Thus, understanding the interactions of both the microbiome and host genetics may provide more insights on disease diagnosis, treatment, and prevention. A number of studies have linked the human microbiome at the various body sites to the development of a wide range of complex traits and diseases, including weight gain, obesity, inflammatory bowel disease, diabetes, cardiovascular disease, cancer, major depression, autism spectrum disorder, and asthma (Kostic et al., 2014; Jiang et al., 2015; Hall et al., 2017; Allali et al., 2018; Qiao et al., 2018). These phenotypic expressions are related to the changes in the overall taxonomic composition, as well as presence or absence of specific bacterial.

Owing to the role played by the microbiome in the pathogenesis of many diseases, there has been a surge of interest in understanding host DNA sequence variations that modulate the human microbiome (Marchesi et al., 2016). Early insights into interaction between host genome and microbiome were obtained from animal based studies (Blekhman et al., 2015; Wang et al., 2016). For example, Rawls et al. (2006) showed that the observed difference between the microbiotas of zebrafish and mice is due to the underlying host genetics. Host genetic loci that shape diversity in skin microbiota and confer susceptibility to disease in mice were also identified (Srinivas et al., 2013). In humans, studies involving monozygotic and dizygotic twins have shown that the abundance of certain microbial taxa are more correlated amongst monozygotic than with dizygotic twin pairs indicating that host genetic factors are involved in modulating gut microbiome composition across human populations (Goodrich et al., 2014; Xie et al., 2016).

The observations above motivated the advent of microbiome genome-wide association study (mGWAS). Using microbiome attributes (such as alpha diversity, beta diversity or relative abundance of bacterial taxa) as the response variable and host’s genotype data as the explanatory variable. mGWAS measures and analyses DNA sequence variations across the host’s genome in order to identify genetic factors that modulate the composition and functional diversity of the microbiome. To date, studies published on mGWAS have raised interest and provided new insights (Blekhman et al., 2015; Davenport et al., 2015; Hua et al., 2015; Bonder et al., 2016; Goodrich et al., 2016; Turpin et al., 2016; Wang et al., 2016; Igartua et al., 2017; Rühlemann et al., 2018; Rothschild et al., 2018). The three first studies using mGWAS were conducted on a relatively small sample size. Blekhman et al. conducted the first mGWAS in 93 individuals using human microbiome data and host genetic information gleaned from the Human Microbiome Project (Gilbert et al., 2010); microbiome data and host DNA were from 15 body sites (Blekhman et al., 2015). The authors identified significant associations between several host genes and pathways with microbiome composition. Following this work, Davenport et al. (2015) reported the second mGWAS which investigated host genetic effects on the gut microbiome of 127 Hutteries (North America) and found host SNPs are associated with the abundance of several bacterial taxa. In the third study, Hua et al. have developed the microbiome-GWAS tool that has been tested on 16S rRNA microbiome data from 147 non-malignant lung tissue samples (Yu et al., 2016) to establish the microbiome composition in terms of cancer risk SNPs. The authors found significant associations between six previously established lung cancer risk SNPs and microbiome composition. Subsequent studies have used larger samples of ∼300–2000 individuals and have reported significant (Bonder et al., 2016; Goodrich et al., 2016; Turpin et al., 2016; Wang et al., 2016). However, Kolde et al. (2018) did not identify significant associations in their untargeted genome-wide analysis in contrast with the findings of Belkhman et al. who used the same cohort and have reported 83 significant associations. Kolde et al. (2018) have reported that the main reason for this difference is the choice of significance thresholds; they used a more stringent Bonferroni correction while Belkhman et al. used false discovery rate (FDR) multiple hypothesis test correction. Additionally, a recent study of 1046 healthy Israeli individuals, with several different ancestral origins and who share a relatively common environment, did not find any significant associations between (Rothschild et al., 2018). The results of the above studies suggest that some bacterial taxa are heritable but the results of one study cannot be replicated except for the bacterial taxa *Bifidobacterium* which were found to be significantly associated with the lactase *LCT* gene locus (Blekhman et al., 2015; Bonder et al., 2016; Goodrich et al., 2016; Rothschild et al., 2018). Table 1 summarizes the mGWAS carried out to date.

**Table 1:** Summary list of microbiome genome-wide association studies (mGWAS) carried out to date.

Study	Year	Sequencing method	Analysis software	Sample size and location	Microbiome sampling site	Microbiome phenotype studied	*N*^o^ of associations identified	Comment
Blekhman et al., 2015	2015	Shotgun metagenomic	PLINK	*n* = 93 United States of America (HMP)	Multiple sites (15)	Alpha diversity, beta diversity, and bacterial taxa	83 associations identified	Host genetic variants correlated with microbiome composition. Variants in the *LCT* gene correlated with abundance of *Bifidobacterium* (*P* = 1.16 (x 10(-5). Genes involved in Leptin signalling pathway significantly associated with microbiome composition; Leptin previously implicated in Obesity.
Davenport et al., 2015	2015	16S rRNA	GEMMA	*n* = 127 Hutterites (North America)	Gut	Bacterial taxa	≥8 bacterial taxa associated with SNPs in host genome in each season	SNPs in regions of the *PLD1* gene associated with abundance of genus *Akkernabsia*; the *PLD1* gene was previously implicated in GWAS of body mass index. one bacterial taxa (genus *Bifidobacterium*) correlated with age. ≥4 bacterial taxa differentially abundant by sex
Hua et al., 2015	2015	16S rRNA	microbiome GWAS	*N* = 147 Italy	Lung	alpha diversity, beta diversity	Six SNPs had suggestive association with beta-diversity	Analysis performed using both weighted and unweighted UniFrac distance matrices. No SNPs were significantly associated after correcting for skewness and kurtosis of beta-diversity distributions.
Goodrich et al., 2016	2016	16S rRNA	microbiome GWAS (for GWAS on the beta diversity measures) GEMMA (for GWAS on taxon)	*n* = 1,126 twin pairs United Kingdom	Gut	Bacterial taxa, beta diversity	31 associated host loci	*LCT* gene associated with *Bifidobacterium*. Also, SNPs in the region of the *R3HDM1* gene strongly associated with *Bifidobacterium* (*P* = 4.38 × 10^-8^). *ALDH1L1* gene associated with SHA-98 bacteria. SNPs associated with beta diversity metrics. alpha diversity found to be heritable. Heritable taxa found, including *Bifidobacterium, Turicibacter*, and *Blautia*.
Bonder et al., 2016	2016	Shotgun metagenomic	“base” in R	*n* = 1,514 Dutch	Gut	Bacterial taxa, bacterial pathways	42 associated host loci	Nine host loci associated with bacterial taxa, and 33 loci with bacterial pathways (*P* < 5 × 10^-8^). *LCT* SNP associated with *Bifidobacterium* (*P* (=3.45 × 10^-8^).
Turpin et al., 2016	2016	16S rRNA	Not sated	*n* = 1,098 (discovery cohort)- Canada and United States *n* = 463 (replication cohort)- Canada, United States and Israel	Gut	Bacterial taxa and alpha diversity	58 suggestive associations only six of which were significantly associated	Of these six SNPs, one was replicated in the replication cohort. Associated taxa included *Rikenellaceae, Faecalibacterium, Lachnospira*, and *Eubacterium*. Many taxa found to be heritable. No significant association with alpha diversity.
Wang et al., 2016	2016	16S rRNA	“envfit” in R	*n* = 1,812 Germany	Gut	Bacterial taxa, beta diversity	54 significant associations	42 loci (which included variants in *VDR* gene -encoding vitamin D receptor) associated with beta diversity (*P* < 5 × 10^-8^). Host loci associated with the *Firmicutes, Proteobacteria*, and *Bacteroidetes*.
Igartua et al., 2017	2017	16S rRNA	GEMMA	*n* = 144 Hutteries (North America)	Vestibule and Nasopharynx sites	Relative abundance (RA) of bacterial taxa, alpha, and beta-diversity	37 significant associations	Most significant association was between variant (rs117042385) upstream of the TINCR gene (*p* (=1.61 × 10^-8^; *q* = 0.002). Differences in RA, alpha and beta diversity were observed across sites and seasons. Significant association between host genetic (measured by kinship coefficient) and microbiome Euclidean distance.
Rühlemann et al., 2018	2017	16S rRNA	“snpStats” in R	*n* (=1,767 Germany	Gut	beta diversity	4four significant associations	The four loci were significantly associated with variation in beta diversity. Reanalysis using permutation-based analysis were still identified all these loci as genome-wide significant.
Kolde et al., 2018	2018	Shotgun metagenomic	Matrix eQTL	*n* (=298 North America (HMP)	Multiple sites (6six)	Relative abundance of bacterial taxa, bacterial pathway	five significant associations with bacterial taxa, and 82 with bacterial pathways	In stool, five species (*Lachnospiraceae bacterium, Roseburia* intestinalis, *Subdoligranulum* (unclassified), *Sutterella wadsworthensis*) out of 118 were significantly associated (false discovery rate (FDR)? <?0.05 by permutation test). In other body sites, *Propionibacterium propionicum, Porphyromonas catoniae*, and unclassified *Gemella* were significantly associated with host genomic variation in buccal mucosa.
Rothschild et al., 2018	2018	16S rRNA and Shotgun metagenomic	“envfit” and “ordiR2step” in R and FaST-LMM	*n* = 1,046 Israel	Gut	Relative abundance of bacterial taxa, alpha, and beta-diversity	seven suggestive associations	No significant association between host genetic variation and bacterial taxa or beta diversity, after correcting for multiple testing.

### mGWAS Approaches and Tools

There are many microbiome attributes that may be leveraged as phenotypes for a mGWAS. First, alpha diversity, that is, the diversity of species within community samples (Wilson and Shmida, 1984) may be used as phenotype and an association performed against host genotypes. Second, beta diversity, that is, the diversity between community samples (Wilson and Shmida, 1984), defined using phylogeny-informed or taxa abundance-informed pairwise distance measures, may be used as phenotype. It is important to note, however, that because the microbiota functions as a community, cross-sample analysis using beta diversity measure is more robust compared to alpha diversity (Hua et al., 2016). Third, the relative abundance of each taxon at a given taxonomic level (species, genus, family, order, class, and phylum) may be used as phenotype, and analysis performed to assess the association of each SNP with the taxon. Alternatively, with shotgun metagenomic sequencing that, unlike the 16S rRNA approach, provides functional information, bacterial pathways may be used as trait for mGWAS. The varied and peculiar features of microbiome phenotypes, particularly, the high dimension (Section “Challenges Underpinning mGWAS”) limit the application of some host GWAS tools. Nonetheless, host GWAS (Zhou and Stephens, 2012) and (Lippert et al., 2011) have been applied in mGWAS using taxonomic abundance as bacterial phenotype; albeit, it cannot be used for association testing with microbiome distance metrics.

To this end, microbiome-specific tool and method have recently been developed: microbiomeGWAS (Hua et al., 2015), and microbiome-association index (Rothschild et al., 2018). microbiomeGWAS uses standard linear regression with beta diversity metrics and corrects for skewness and kurtosis. It identifies host genetic variants associated with microbiome beta diversity by testing both SNP-microbiome and SNP-environment interactions. Because the statistical power of these distance-based measures depends on the choice of the distance metric, this tool was subsequently improved to accommodate multiple distance matrices. Meanwhile, microbiome-association index (*b*^2^) has been specifically developed to quantify the overall association of microbiome to host’s phenotype, incorporating the contribution of host genetics. Using this association index, a measure similar to narrow sense heritability in hGWAS, the authors showed that several host phenotypes, including body mass index, fasting glucose levels, glycaemic status, and lactose consumption, exhibited substantial *b*^2^ values in the range of 22–36%. In addition, different statistical methods have been used in mGWAS such as the ordination and permutation-based envfit, ordi2step, snpStats, and Spearman’s correlation statistical methods (Table 1).

## Disparity in Host and Microbiome GWAS

Despite the meteoric rise in GWASs over the last few years, the number of studies inclusive of genetically diverse populations is disproportionately low. A 2015 assessment of the number of NIH-funded GWAS focused on or utilizing non-European populations revealed great disparity; for example, of the 4,942 publications, African American, Hispanic, and Jewish ancestry constituted only ∼3%, <1%, and <1%, respectively, of the sampling study population (Peprah et al., 2015). A recent analysis of a curated database of genomic variants associated with various traits/diseases, provided by the National Human Genome Research Institute (NHGRI) and the European Bioinformatics Institute (EMBL-EBI), revealed a bias in genomic diversity and disproportionate representation (in terms of ancestry, and physical and social environments of the study subjects) in published GWAS (Hindorff et al., 2018). As of August 2016, non-European ancestry represented only 19% of all individuals in GWAS. This becomes a pertinent issue given the observation that non-European individuals contribute a larger number of genotype-phenotype associations (Morales et al., 2018; Hindorff et al., 2018), and studies in other (non-European) population groups continue to identify novel genetic variants. This disparity in population representation is not confined to host GWAS. Although a relatively young field, mGWAS carried out since its inception in 2015, show marked disparity in terms of genetic diversity and population representation. As depicted in Figure 2, most studies involved individuals from North America and Europe. With mGWAS findings differing markedly across all these studies, better inclusivity of diverse populations will illuminate any underlying interactions since it is possible that the interaction of host genetic variant(s) with the microbiome may be population-, environment-, or even individual-specific owing to other yet-unknown clinical and environmental factors.

From a statistical genetics perspective, while the inclusion of non-European populations, in particular the African population, is a critical step toward discovery of important host–genetic interactions in traits/diseases, the implementation requires methodological and technological refinement. It is well known that variants associated with diseases found in populations of European descent do not always replicate in non-European, particularly African populations (Peprah et al., 2015). This discrepancy across populations are due to several possible reasons including differences in allelic architecture, LD, and environmental factors across populations (Popejoy and Fullerton, 2016; Bentley et al., 2017). Thus, there is a need to design appropriate novel statistical models that are tailored to leverage the characteristics of non-European subjects. Moreover, most of the current technologies for mining genetic data, for example genotyping arrays, have been designed for populations of European descent with long-range patterns of LD (Peprah et al., 2015; Popejoy and Fullerton, 2016) or nearly homogeneous environments.

## Challenges Underpinning mGWAS

Microbiome genome-wide association studies findings have provided unprecedented views into the association of human host genes with microbes or microbial genes. However, there are several key challenges which also present new opportunities that need to be tackled if we are to assemble a global understanding of host-genetic association with the microbiome.

### Demographic and Environmental Factors

The human microbiome is sensitive to a wide range of demographic and environmental factors. Factors as diverse as gender (Foster et al., 2017), age (Mäkivuokko et al., 2010), and geography (Yatsunenko et al., 2012) have all been shown to influence the composition and functional diversity of the microbiome. These factors can introduce sampling artifacts or biases in mGWAS which can reduce statistical power. Therefore, the statistical models need to adjust for, the effect of these factors. This is particularly important if such factors do not have interaction effect with the genetic variants. Accounting for these factors remains a fundamental challenge for mGWAS. Although it is nearly impractical to adjust for all these factors in a typical setting, it is imperative that enumerable factors be considered as covariates in downstream analyses. Adjustment for potential endogenous and exogenous sources of variability will be key aspects for providing reliable and replicable results.

### The Complexity of Microbiome Data

Pertinent to mGWAS, the complexity of microbiome data in terms of dimension, phenotype and correlation structure presents a challenge in the development of robust association frameworks. Microbiome data is highly dimensional, often consisting of hundreds of bacterial taxa. When searching for genetic variants associated with a bacterial trait, multiple tests are carried out, each time testing the null hypothesis of no difference in genotype distribution. With many taxa, this leads to not only high computational cost, but increases in the number of statistical tests. This requires correction for multiple testing to control for the occurrence of false positives. The correction (commonly genome-wide significance, permutation tests, FDR or Bonferroni correction), however, introduces yet another challenge – a potential reduction in statistical power – especially when an underpowered adjustment procedure or an inappropriate error rate is used. The inherent strengths and limitations of each of these correction methods influence association results. For example, (Blekhman et al., 2015) and (Kolde et al., 2018), using FDR and Bonferroni corrections, respectively, obtained contrasting results using the same research cohort. The current solution to circumvent the issue of multiple testing is to focus on only a subset of taxa or variants. For example, (Davenport et al., 2015) reduced the number of bacterial taxa by removing all taxa highly correlated with taxa at the same or lower taxonomic level. Meanwhile, in another study by Bonder et al. (2016), the authors performed a targeted association analysis focusing only on SNPs in genes related to immunity and metabolism. While robust in detecting true associations, using only a selection of taxa leads to incomplete representation of microbiome composition and, consequently, limits the opportunity to discover novel associations. This issue is critical as there may be specific rare taxa that can interact with the host genetics. Likewise, although powerful at dissecting association at a biologically plausible region of interest, reducing the number of host genes variants examined can result in exclusion of relevant genes from the analysis.

In addition, the genetic architecture, the landscape of contributions of host genetics to a given microbiome phenotype, is at best poorly understood although studies of host–genetic interactions with microbiome suggests polygenicity. Moreover, the reported percentage of microbiome variation explained by the associated alleles are generally very small (for example 0.65–0.97% in Wang et al., 2016), and thus unable to explain much of the variability in microbiome phenotype. Given this effect size, large sample sizes will be required to detect modestly associated variants. Meanwhile, the high level of trait collinearity coupled with complex correlation structure (Kurilshikov et al., 2017) also make it challenging for statistical methods. Even though parametric linear models remain the cornerstone for genetic association studies and have played pivotal role in mGWAS carried out to date, they are limited to detecting non-linear interaction patterns. In particular, when modeling complex structures such as varying effect and non-linear interactions, the exponential rise in the number of parameters increases computational cost and reduces statistical power (Moore et al., 2010). Moreover, linear models generally treat interaction effects as factors with independent marginal effects; a strategy that lowers its power in the presence of interaction effects (Millstein et al., 2006). Given these limitations, there is a need to develop mGWAS-adapted statistical methods to complement existing linear models.

### Replicability of Results

Besides the challenges toward achieving reliable results, replication of mGWAS results has been poor. The first mGWAS, (Blekhman et al., 2015), using 93 individuals and bacterial taxa as phenotypes, detected 83 associations between genetic polymorphisms in host coding genes and the abundance of specific bacterial taxa. Of note, was the association of immune-related genes, *HLA-DRA* and *TLR1*, with abundance of *Selenomonas* and *Lautropia*, respectively, and the strong link between SNPs in the lactase persistence gene, *LCT*, and abundance of *Bifidobacterium*. In subsequent studies, for example, (Davenport et al., 2015; Bonder et al., 2016; Goodrich et al., 2016), many immune and metabolism-related host genes were found to be significantly associated with abundance of bacterial taxa and beta diversity measures. However, there was little congruence between the results across these studies. This can possibly be attributed to factors including differences in statistical methods, multiple-testing corrections, lifestyle, diet, demographic and environmental conditions of the samples. Nonetheless, the enrichment of microbiome-associated variants with immunity and metabolism related genes, and the generally small effect size, the percentage of microbiome variation explained by the genes, (<1%) remain the most consistent. This suggests that a large proportion of heritability for most bacterial traits may be accounted for by many small effect genetic polymorphisms in immunity and metabolism-encoding genes. A corollary is that bacterial traits likely have an infinitesimal genetic architecture, requiring meta-analyses to detect the associated variants. A combination of larger sample sizes, unified robust analysis methods, and inter-cohort analyses facilitated by collaborations such as MiBioGen consortium (Wang et al., 2018) are crucial for both the attainment of power to detect small to moderate host genetic effects on microbiome traits and replicability of findings.

## Conclusion and Perspectives

**FIGURE 3:**
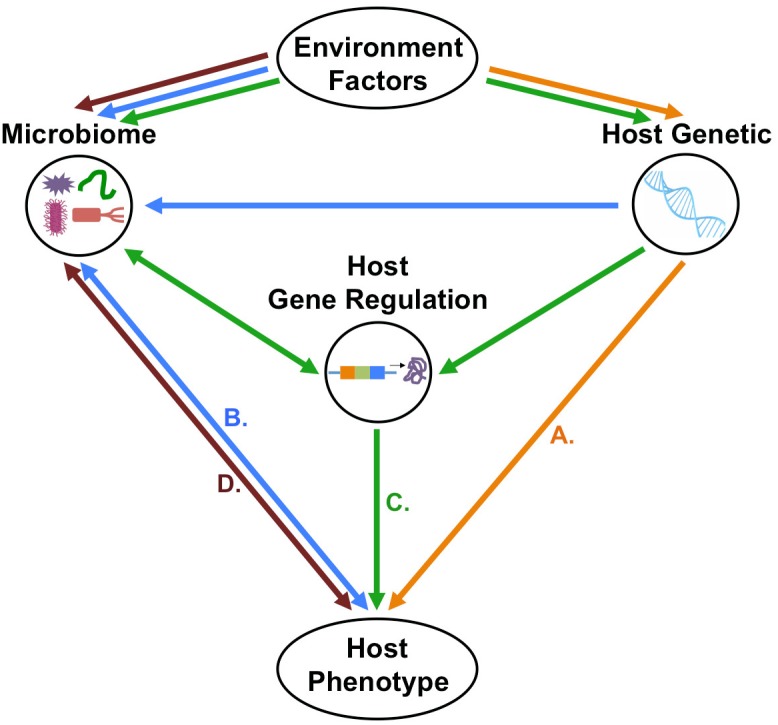
Possible direction of host–microbiome–environment interactions in the context of host phenotypes. (A) First possibility is that host-genetic polymorphism with or without the environmental effects will influence host phenotype independently of host–microbiome interactions. (B) Second possibility is that host genetic polymorphisms do not directly determine phenotype, but rather, host–microbiome interactions and environmental factors modulate the microbiome, which, in turn shapes the host phenotype. (C) Third possibility is that host genetic variation and microbiome changes, both influenced by environmental factors, affect host gene regulation which will control the host’s phenotype. (D) Fourth possibility that is the microbiome–environment interactions will directly affect host phenotype independently of host genetic.

Discovery of the role of the microbiome in normalcy and disease status spurred efforts to elucidate the interaction of host genetic variation with the microbiome, leading to the development of mGWAS. Initial foray into mGWAS has led to the discovery of host genetic variants that contribute to variability in compositional and functionality diversity of the microbiome. Despite the significant strides made in this field, further developments are still needed to elucidate the extent, direction, and mechanism of host-microbiome association and how this association ultimately impact on host’s phenotypic expression (Figure 3).

Moving forward, the importance of sufficient sample sizes cannot be overstated. This will require a collaborative approach, pooling samples from across different geographic regions of the world to generate sufficiently powered studies for discovery and replication. In doing so, the trade-off between sample size and between-sample heterogeneity must be carefully assessed, given the myriad of factors that can reduce the association power of mGWAS. Otherwise, a wide between-sample differences resulting from temporal and spatial heterogeneities will reduce the power to detect true association. In addition to sample size, maximizing bacterial trait information will potentially increase detection power. Current mGWAS has focused on independent analysis of various bacterial traits. This is probably due to the current lack of known software applications that can enable a joint analysis of microbial taxa/pathway and microbial diversity. Given the relative etiological similarity of these traits, it is likely that such joint multiple phenotype analysis will maximize discovery power.

**FIGURE 4:**
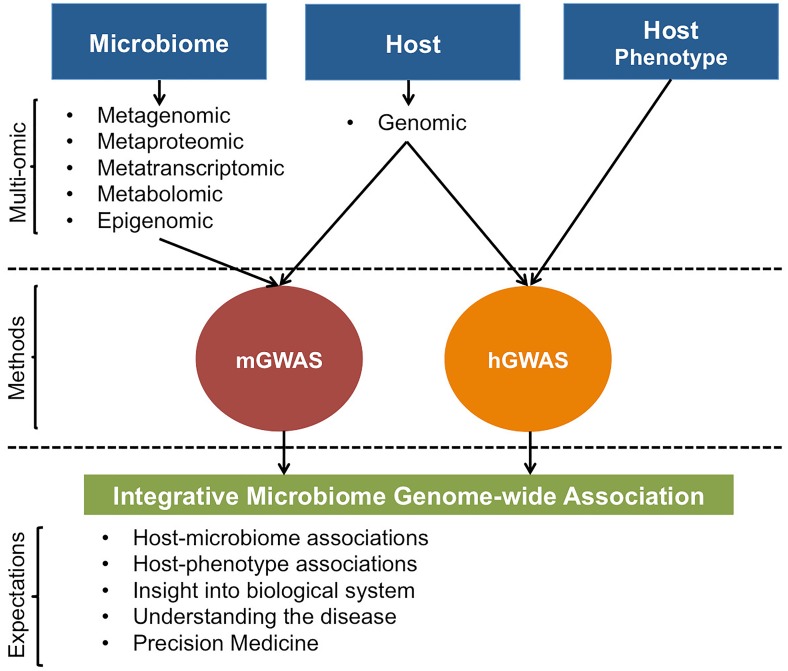
Illustrative representation of possible host and microbiome GWAS approaches. For mGWAS, different microbiome omic data could be individually or jointly regressed with host genomic data. Results from mGWAS and hGWAS will clarify on host-microbiome associations, effect of host-microbiome associations on the phenotype, and provide insight into biological system by giving a better view of the interaction networks that underlie expression of host phenotypes.

In addition, even though mGWAS to date have primarily focused on the genomic level, regressing host’s genetic variation with microbiome’s transcriptomic, metabolomic, and proteomic data types or integrating them in a joint host genotype-microbiome association analysis (Figure 4) will be an exciting venture. mGWAS using these multi-level data types can potentially yield insights into whether host–microbiome interactions are, if any, universal or more pronounced to a specific microbial attribute; facilitating identification of particular host genetic polymorphisms that interacts with the microbiome on a molecular level. This is crucial if mGWAS results are to have any utility at level of understanding host’s clinical and biological states.

Furthermore, the integration of environmental factors in mGWAS will lead to an exciting starting point and perspective for a more comprehensive and robust analysis of host-microbiome interaction in more powered studies. However, the current challenges include difficulty of adjusting for potential environmental factors, complexity of microbiome data, and lack of robust and unified analytical frameworks to handle the diverse and peculiar properties of microbial attributes as quantitative traits. Together, addressing these challenges coupled with increased sample size, independent replication, and meta-analysis in multiple populations will provide a more complete understanding of human variation and microbial diversity in connection with health and disease.

## Author Contributions

EC, DA, and IA conceived and structured the manuscript. DA, IA, SD, SH, KM, NT, AG, NM, and EC generated the contents and wrote the manuscript.

## Conflict of Interest Statement

The authors declare that the research was conducted in the absence of any commercial or financial relationships that could be construed as a potential conflict of interest.
